# SYBR green-based one step quantitative real-time polymerase chain reaction assay for the detection of Zika virus in field-caught mosquitoes

**DOI:** 10.1186/s13071-017-2373-4

**Published:** 2017-09-19

**Authors:** Wei-Ping Tien, Gareth Lim, Gladys Yeo, Suzanna Nicole Chiang, Chee-Seng Chong, Lee-Ching Ng, Hapuarachchige Chanditha Hapuarachchi

**Affiliations:** 1 Environmental Health Institute, National Environment Agency, 11, Biopolis Way, #06-05-08, Singapore, 138667 Singapore; 2 School of Biological Sciences, Nanyang Technological University, 60 Nanyang Drive, Singapore, 637551 Singapore

**Keywords:** Zika virus, RT-PCR, Vector, Diagnostics, Surveillance

## Abstract

**Background:**

The monitoring of vectors is one of the key surveillance measures to assess the risk of arbovirus transmission and the success of control strategies in endemic regions. The recent re-emergence of Zika virus (ZIKV) in the tropics, including Singapore, emphasizes the need to develop cost-effective, rapid and accurate assays to monitor the virus spread by mosquitoes. As ZIKV infections largely remain asymptomatic, early detection of ZIKV in the field-caught mosquitoes enables timely implementation of appropriate mosquito control measures.

**Results:**

We developed a rapid, sensitive and specific real-time reverse transcription polymerase chain reaction (rRT-PCR) assay for the detection of ZIKV in field-caught mosquitoes. The primers and PCR cycling conditions were optimized to minimize non-specific amplification due to cross-reactivity with the genomic material of *Aedes aegypti*, *Aedes albopictus*, *Culex quinquefasciatus*, *Culex tritaeniorhynchus*, *Culex sitiens* and *Anopheles sinensis*, as well as accompanying microbiota. The performance of the assay was further evaluated with a panel of flaviviruses and alphaviruses as well as in field-caught *Ae. aegypti* mosquitoes confirmed to be positive for ZIKV. As compared to a probe-based assay, the newly developed assay demonstrated 100% specificity and comparable detection sensitivity for ZIKV in mosquitoes.

**Conclusions:**

Being a SYBR Green-based method, the newly-developed assay is cost-effective and easy to adapt, thus is applicable to large-scale vector surveillance activities in endemic countries, including those with limited resources and expertise. The amplicon size (119 bp) also allows sequencing to confirm the virus type. The primers flank relatively conserved regions of ZIKV genome, so that, the assay is able to detect genetically diverse ZIKV strains. Our findings, therefore, testify the potential use of the newly-developed assay in vector surveillance programmes for ZIKV in endemic regions.

**Electronic supplementary material:**

The online version of this article (10.1186/s13071-017-2373-4) contains supplementary material, which is available to authorized users.

## Background

Zika virus (ZIKV) is an arbovirus that was first isolated in 1947 from a rhesus monkey in Uganda and was subsequently identified in *Aedes africanus* mosquitoes in 1948 [1]. Although the first authentic ZIKV infection in humans was described in early 1960s [2], ZIKV remained dormant until 2007 when it re-emerged in Micronesia [3]. The re-emergence has caused a new wave of epidemics in Africa, Asia, islands of the tropical Pacific Ocean and the Americas [4]. Despite being associated with complications such as microcephaly and Guillain-Barré syndrome, a large proportion of ZIKV infections are asymptomatic and self-limiting [4], thereby, is silently transmitted and underreported. ZIKV is transmitted primarily through the bites of *Aedes* mosquitoes, which also transmit dengue (DENV) and chikungunya viruses [5]. As *Aedes* mosquitoes are widely distributed, there is an imminent threat of recurrent ZIKV epidemics, especially in the tropics and subtropics.

Singapore reported the first indigenous outbreak of ZIKV in August 2016 [6]. Due to the widespread presence of *Aedes* mosquitoes, high human density and lack of population exposure to ZIKV, there was a substantial threat of an island wide epidemic and sustained transmission. Vector control is the cornerstone in controlling ZIKV transmission, and the National Environment Agency, Singapore stepped up the vector surveillance and control activities to avert further virus spread in the country. One of the main components of the vector surveillance activities was to screen vector populations for the presence of ZIKV in order to monitor the risk of sustained transmission and to prioritize resource allocation for vector control operations.

The laboratory detection of ZIKV mainly relies on the detection of virus RNA by molecular methods such as polymerase chain reaction (PCR) and related technologies; e.g. loop mediated isothermal amplification [7–11]. The majority of available assays are probe-based real-time reverse transcription PCR (rRT-PCR) methods. While probe-based rRT-PCR protocols tend to be more sensitive and specific than SYBR Green-based protocols, the latter is more cost-effective and easier to adopt from published primer sequences. This makes SYBR Green-based methods one of the preferred choices for PCR-based mass screening purposes in disease surveillance programmes, especially in resource-poor settings that also lack expertise in assay design and optimisation. Moreover, SYBR Green-based methods allow the amplification of longer PCR fragments than probe-based protocols, thus enable sequencing a reasonable length of genomic fragments to affirm the pathogen/s, especially in non-endemic regions. Nevertheless, only a handful of SYBR Green-based RT-PCR methods have been published for the detection of ZIKV RNA [8, 11].

One of the main limitations in using the available methods for the vector surveillance is that they have primarily been designed for ZIKV detection in human biological samples [9, 12, 13]. The molecular assays optimised for human biological samples may not necessarily perform in a similar manner in field-caught mosquito samples, potentially due to complex nature of inherent and microbiota genomic composition that may affect the assay specificity [14]. None of the methods described thus far, except for that of Faye et al. [10], has compared the assay performance in field-caught mosquitoes. In order to support ZIKV surveillance in mosquito populations, we developed a SYBR Green-based RT-PCR assay optimized for the detection of ZIKV in mosquitoes and compared its performance with one of the probe-based assays [9] in field-caught mosquitoes.

## Methods

### Designing of primers

Whole genome sequences of all genotypes of ZIKV were retrieved from the GenBank nucleotide database and aligned using the BioEdit software [15]. The alignment was used to map already-published ZIKV-specific primers [9] and to design new primers in adjoining upstream and downstream conserved regions overlapping the membrane and envelope genes (Table 1).

**Table 1:** Details of the primer sequences and combinations evaluated for the assay development

Primer name	Sequence (5′-3′)	Genome position^a^
Forward primers		
ZIKV-F^b^	TTGGTCATGATACTGCTGATTGC	941–963
ZIKV-F2	TCAACGAGCCAAAAAGTCAT	917–936
ZIKV-F3	ATCAGGTGCATAGGAGTCAG	977–996
Reverse primers		
ZIKV-R^b^	CCTTCCACAAAGTCCCTATTGC	996–1016
ZIKV-R3	CAGGTCCCACCTGACATGC	1017–1035
ZIKV-R4	CAACGACTGTCCGAAGCCATG	1177–1197

^a^Genome positions were calculated from the first base of the 5′ non-translated region of the ZIKV reference sequence in GenBank nucleotide database (Accession no. NC012532.1)

^b^Primers previously used for a probe-based rRT-PCR protocol published by Lanciotti et al. [9]

### Viruses

ZIKV strains ATCC® VR-84 (GenBank: NC012532.1) and PLCal_ZV (GenBank: KF993678) provided by Public Health Agency of Canada [16] were used to prepare viral stocks. Viral stocks were prepared by inoculating each isolate into Vero cells (African Green Monkey Kidney) in M199 growth medium (Invitrogen, Carlsbad, CA, USA) supplemented with 10% foetal bovine serum, 1% L-glutamine solution and 100 mM Penicillin/Streptomycin (Sigma-Aldrich, St. Louis, MO, USA). After five days of propagation, viral titres were quantified by using the plaque assay performed on BHK-21 cells as described elsewhere [17].

### Extraction of viral RNA and mosquito-derived RNA

Viral RNA was extracted from known titres of viral stocks by using the QIAamp viral RNA mini kit (Qiagen, Hilden, Germany) according to manufacturer’s instructions. All primers were initially optimised using ATCC® VR-84 RNA for the amplification efficiency and annealing temperature.

Virus stocks of both ATCC® VR-84 and PLCal_ZV strains were 9-fold diluted to obtain virus titres from 10^4^ to 10^−4^ PFU/ml. Pools (five mosquitoes per pool) of laboratory-strains of *Aedes aegypti*, *Aedes albopictus* and *Culex quinquefasciatus* mosquitoes in 1× phosphate-buffered saline were spiked with serial dilutions of viruses. In order to evaluate the primer cross-reactivity with RNA derived from wild mosquitoes, we included field-caught *Ae. aegypti*, *Ae. albopictus*, *Culex tritaeniorhynchus*, *Culex sitiens* and *Anopheles sinensis* adult mosquitoes that were screened negative for flaviviruses, using a pan-flavivirus PCR assay [18]. These samples were not spiked with ZIKV RNA before RNA extraction. Spiked and non-spiked mosquito samples were homogenized in a mixer mill (Retsch, Haan, Germany) as described before [19]. RNA was extracted from 140 μl of each homogenate by using the QIAamp viral RNA mini kit (Qiagen, Hilden, Germany) according to manufacturer’s instructions. The quantification of RNA extracted from non-spiked field-caught mosquitoes is given in Additional file 1: Table S1. The amount of mosquito-derived RNA varied based on the species as well as tissues used (whole mosquitoes versus head and thorax only) and reached levels similar to RNA extracted from virus cultures (> 1000 ng/ml). Forty units of RNaseOUT (Life Technologies, Carlsbad, CA, USA) were added to each elute to minimize RNA degradation. RNA extracted from non-spiked samples was mixed with an equal volume of ZIKV (PLCal_ZV strain, 10^4^ PFU/ml) RNA to test for the cross-reactivity of primers in the presence of genomic material derived from mosquitoes.

### Collection of field mosquitoes in a ZIKV cluster

Adult mosquitoes were caught using Gravitraps [20] between epidemiological week (EW) 34 of 2016 and EW12 of 2017 to monitor ZIKV transmission in the first zika fever cluster in Singapore [6]. The field-caught mosquitoes were first identified to species level before being excised individually into two parts: abdomen and head/thorax. One to five abdomens were pooled before the extraction of RNA using the QIAamp viral RNA mini kit (Qiagen, Hilden, Germany) according to the manufacturer’s instructions. The mosquito pools were screened for ZIKV by using a probe-base rRT-PCR assay as described elsewhere [9]. The head and thorax samples of mosquitoes in positive abdomen pools were screened individually by using the same rRT-PCR assay to determine the extact location of virus transmission.

### SYBR green-based real time RT-PCR assay

One-step rRT-PCR assay was carried out using QuantiTect SYBR Green RT-PCR Kit (Qiagen, Hilden, Germany) in a 20 μl reaction consisting of 5 μl of extracted ZIKV RNA and 1 μl of each primer mix (Table 2) in a LightCycler® 2.0 (Roche Diagnostics GmbH, Mannheim, Germany) machine. The following thermal profile was used: reverse transcription at 50 °C for 20 min and inactivation at 95 °C for 15 min, followed by 40 cycles of 94 °C for 15 s, 52–55 °C for 30 s and 72 °C for 30 s. The exact annealing temperatures used for each primer pair is given in Table 2. A melting curve analysis was performed at 65 °C for 15 s followed by a cooling step at 37 °C for 20 s to verify the accuracy of amplified products. The amplicon sizes were confirmed by 2% agarose gel electrophoresis. A standard curve for the quantification of viral titres was obtained by plotting quantification cycle (C_q_) values against known virus titres for each dilution. The amplification efficiency and coefficient of determination (R^2^) were calculated using Microsoft Excel (Microsoft, Redmond, WA, USA).

**Table 2:** Primer combinations evaluated during the assay development

Primer combination	Amplification success	Product size (bp)
F + R	Successful amplification; annealing at 55 °C	76
F + R3	Successful amplification; annealing at 55 °C	95
F + R4	Successful amplification; annealing at 55 °C	257
F2 + R	Successful amplification; annealing at 52 °C	100
F2 + R3	Successful amplification; annealing at 52 °C	119
F2 + R4	Successful amplification; annealing at 52 °C	281
F3 + R	Failed amplification	40
F3 + R3	Failed amplification	59
F3 + R4	Successful amplification; annealing at 52 °C	221

### Performance parameters of the assay

The specificity of the assay for ZIKV was determined by using RNA extracted from a panel of flaviviruses; DENV-1 (GenBank: KM403575), DENV-2 (GenBank: KR779784), DENV-3 (GenBank: KR779787), DENV-4 (GenBank: KR779790), Japanese encephalitis virus (Nakayama Strain, courtesy of Dr. Y.C. Chan), West Nile virus, Kunjin virus, yellow fever virus (17D vaccine strain), and alphaviruses; Ross River virus, Sindbis virus and chikungunya virus (GenBank: KY883764).

## Results

### Assay design and optimisation

The published [9] and newly designed primers were permutated to evaluate the performance of primer mixes that generate different amplicon sizes. Of nine primer mixes tested, only seven pairs were able to yield successful amplification (Table 2). We further evaluated the sensitivity and specificity of four different primer pairs (ZIKV-F + R3, F + R4, F2 + R3, and F3 + R4) that amplified a range of amplicon sizes (76–257 bp). Longer amplicon sizes were preferred because of their suitability for sequencing to determine virus lineages.

**Fig. 1:**
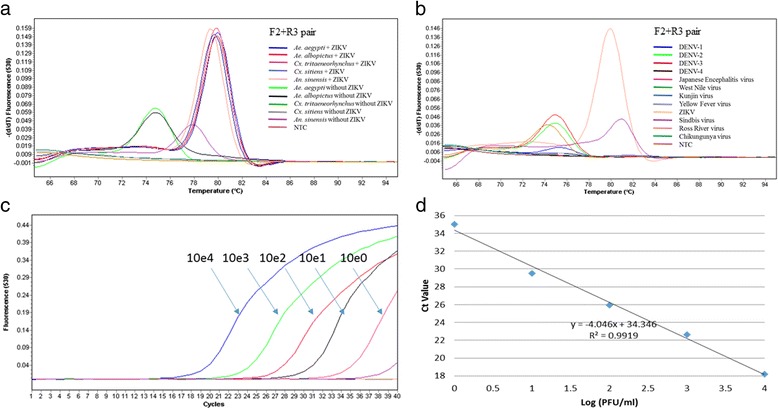
Performance of the rRT-PCR assay utilising the primer combination F2 + R3. **a** Specificity of the assay in the presence of mosquito RNA. The mosquito RNA was spiked with a 5-fold dilution (10^2^–10^-2^ PFU/ml) of ZIKV RNA, to determine any primer cross-reactivity with mosquito-derived genomic material. **b** Specificity of the assay in a panel of flaviviruses and alphaviruses. The melting peak for ZIKV ranged between 80.3–80.4 °C, while that of primer dimers ranged between 73.0–75.8 °C. **c** Amplification curves for 10-fold serial dilutions (10^4^–10^-4^ PFU/ml) of ZIKV. **d** Standard curve for the quantification of ZIKV infectious virus particles. The curve was generated by plotting the quantification cycle (C_q_) values against the log10 of infectious virus particles (PFU/ml). Only the detection range is shown. RNA from ZIKV isolate PLCal_ZV was used in the analysis

To determine the assay specificity in the presence of mosquito RNA, the four different primer pairs (ZIKV-F + R3, F + R4, F2 + R3, and F3 + R4) were first tested with RNA extracted from mosquito pools spiked with ZIKV (Table 3) and pools that were not spiked with ZIKV RNA (Additional file 2: Table S2). This step also determined any background signal generated due to primer cross-reactivity with mosquito-derived RNA. Among the four combinations tested, ZIKV-F + R4 and ZIKV-F3 + R4 primer pairs demonstrated substantial cross-reactivity (Additional file 3: Figure S1a, b). Therefore, ZIKV-F + R3 (Additional file 3: Figure S1c) and ZIKV-F2 + R3 (Fig. 1a) combinations were shortlisted for further evaluation of the cross-reactivity and specificity in a panel of flavivirus and alphavirus RNA as well as with mosquito-derived RNA. None of the eight flaviviruses tested gave positive results for both primer pairs (Fig. 1b; Additional file 3: Figure S1d). However, two alphaviruses, namely Sindbis virus (T_m_ = 80.07, C_q_ = 30.24) and Ross River virus (T_m_ = 80.02, C_q_ = 27.09) gave positive signals for ZIKA-F + R3 (Additional file 3: Figure S1d), but not for the ZIKV-F2 + R3 primer mix (Fig. 1b). Therefore, ZIKV-F2 + R3 primer pair was selected for the further evaluation.

**Table 3:** Comparison of the sensitivity of ZIKV detection between a probe-based rRT-PCR assay and the SYBR Green-based assay utilising the primer combination F2 + R3

ZIKV strain^b^	Mosquito species	Detection threshold of virus titre (PFU/ml)	
		Probe-based rRT-PCR^a^	SYBR Green-based rRT-PCR
ATCC® VR-84	*Aedes aegypti*	100	10
	*Aedes albopictus*	10	1
	*Culex quinquefasciatus*	10	100
PLCal_ZV	*Aedes aegypti*	1	1
	*Aedes albopictus*	0.1	0.1
	*Culex quinquefasciatus*	10	10

^a^The assay protocol has previously been described by Lanciotti et al. [9]

^b^ZIKV strain ATCC® VR-84 is the prototype strain isolated in Uganda in 1947 (NCBI accession No. NC012532.1). The strain belonging to African genotype. PLCal_ZV strain was isolated in a Canadian traveller from Thailand in 2013 (NCBI accession No. KF993678). PLCal_ZV belongs to the Asian genotype

### Evaluation of sensitivity

In the absence of mosquito RNA, the detection sensitivity of the assay ranged between 10^4^ and 10^0^ PFU/ml (Fig. 1c). The C_q_ values of ZIKV-F2 + R3 primer pair showed a linear curve (*R* ^2^ = 0.99) for the detection of ZIKV (Fig. 1d) and the standard curve generated allows quantification of infectious virus particles based on C_q_ values. In order to determine the ZIKV detection sensitivity in the presence of mosquito RNA, we used a 5-fold serial dilution (10^2^–10^-2^ PFU/ml) of the total RNA extracted from pools of mosquitoes spiked with ZIKV (10^4^ PFU/ml). The sensitivity of detection of ZIKV RNA in the presence of *Ae. aegypti*, *Ae. albopictus* and *Cx. quinquefasciatus* RNA was 10, 1, and 100 PFU/ml, respectively, indicating at least one log drop of sensitivity in the presence of mosquito RNA (Table 3). Of two reference strains tested, the assay generated better detection sensitivity for the Asian genotype of ZIKV (PLCal_ZV) (Table 3), which is attributable to the recent emergence of ZIKV in the Americas and Asia [4]. Overall, the detection sensitivity of the newly developed assay for ZIKV in mosquitoes was comparable to that of a probe-based assay described previously [9] (Table 3).

### Evaluation of the assay in ZIKV infected field caught mosquitoes

To determine the applicability of the SYBR Green rRT-PCR assay for ZIKV surveillance in field-caught mosquitoes during disease outbreaks, we screened a panel of *Ae. aegypti* mosquitoes collected from the first local ZIKV outbreak cluster [6] (Table 4). The panel included seven head/thorax samples that were confirmed to be positive for ZIKV by the probe-based assay described by Lanciotti et al. [9]. These ZIKV-positive mosquitoes were collected as part of the vector surveillance activities carried out during the outbreak. We also included a similar number of ZIKV-negative mosquito RNA samples to determine the specificity. The results, summarized in Table 4, showed that the SYBR Green assay was 100% specific and produced either an equal or better sensitivity profile as compared to the probe-based assay. The results also testified the absence of cross-reactivity with the genomic material of field-caught mosquitoes (Additional file 4: Figure S2).

**Table 4:** Evaluation of SYBR Green-based rRT-PCR assay performance in ZIKV infected field caught mosquitoes confirmed by a probe-based rRT-PCR assay

Sample ID^b^	Probe-based rRT-PCR^a^		SYBR Green-based rRT-PCR	
	C_q_ value	Result	C_q_ value (T_m_)	Result
HT55	21.06	Positive	17.57 (80.38)	Positive
HT66	19.54	Positive	15.23 (80.24)	Positive
HT67	28.77	Positive	26.06 (80.16)	Positive
HT68	29.19	Positive	26.98 (80.22)	Positive
HT74	17.09	Positive	13.74 (80.42)	Positive
HT81	19.66	Positive	16.33 (80.1)	Positive
HT83	33.23	Positive	35 (80.04)	Positive
*Ae. albopictus*	Not available	Negative	Not available	Negative
*Ae. albopictus*	Not available	Negative	Not available	Negative
*Culex* spp.	Not available	Negative	Not available	Negative
*Culex* spp.	Not available	Negative	Not available	Negative
*Ae. aegypti*	Not available	Negative	Not available	Negative
*Ae. aegypti*	Not available	Negative	Not available	Negative
*Ae. aegypti*	Not available	Negative	Not available	Negative
ZIKV positive control (ATCC® VR-84)	16.77	Positive	13.84 (79.88)	Positive
Negative control	Not available	Negative	Not available	Negative

*Abbreviations*: *C*_*q*_ quantification cycle, *T*_*m*_ melting temperature

^a^The assay protocol has previously been described by Lanciotti et al. [9]

^b^Sample IDs from HT55 to HT83 are *Ae. aegypti* specimens collected from a zika fever cluster during the first outbreak of ZIKV in Singapore in August 2016 [6]. They were confirmed to be ZIKV-positive by the probe-based rRT-PCR assay. ZIKV negative mosquito specimens collected from the field, including the same cluster, are included for the comparison of specificity

## Discussion

Screening of both human and vector populations for the presence of pathogens is equally important to vector-borne disease surveillance programmes. First, the data obtained from vector populations enables the confirmation of autochthonous transmission, in the event of emergence/re-emergence of non-endemic pathogens. The data also helps to monitor the subsequent expansion of pathogen transmission. The longitudinal monitoring of vector populations, especially in disease clusters, signals whether the transmission is sustained or interrupted, and thereby the successfulness of control measures in place.

Pathogen-specific molecular assays are unarguably applicable to both human and vector samples. However, as compared to human biological samples, vector specimens tend to generate higher “background genetic noise” due to the presence of host as well as other related organisms in the microbiome. The potential “cross-talk” between the primers and non-targeted template sites may influence the sensitivity, specificity and efficiency of PCR amplification. Therefore, assays optimized for human samples may need to be modified, re-developed and evaluated before being used for pathogen surveillance in vector populations. This is notably applicable to assays with detection mechanisms that tend to be less specific. For example, SYBR Green intercalates with any double-stranded DNA product and emits a positive signal. Consequently, non-specific amplifications may be captured as false positives. This is in contrast to probe-based assays that require the template annealing of a specific, short genomic sequence to generate a positive signal, thereby maintaining a high level of specificity. Therefore, SYBR Green rRT-PCR assays need extra diligence in primer designing and protocol optimization to achieve comparable performance to probe-based assays [21, 22]. As demonstrated in the present study, we tested several combinations of primers that were either adapted from a previously published protocol [9] or newly-designed within the same conserved region of ZIKV genome. Many of them generated non-specific, background noise that was difficult to be differentiated from a true signal in the SYBR Green assay format. However, ZIKV-F2 + R3 primer combination exhibited negligible level of cross-reactivity with a panel of different mosquito species, alphaviruses and flaviviruses, while maintaining detection sensitivity comparable to a known probe-based assay for ZIKV [9].

Despite the common drawback of being less-specific than probe-based assays, SYBR Green technology is widely used as a cheap alternative for the rRT-PCR-based pathogen detection [21]. The cost benefit of SYBR Green approach justifies its applicability in mass screening exercises, such as surveillance programmes. The newly-developed SYBR Green assay showed 100% sensitivity and specificity in a panel of ZIKV-positive mosquitoes collected from a disease cluster, demonstrating its suitability for vector surveillance programmes. Importantly, the assay demonstrated higher sensitivity of detection for the Asian genotype of ZIKV, which is attributed to the recent emergence of zika fever in the Americas and Asia [4]. Even though the assay was optimized by using two ZIKV strains of both Asian and African genotypes, the primers flank relatively conserved regions of ZIKV genome, indicating the universal applicability of our assay for the detection of genetically diverse ZIKV strains. Our findings, therefore, support the potential use of newly-developed assay for the screening of field-caught mosquitoes as part of ZIKV surveillance programmes in endemic regions.

## Conclusions

Recent emergence and inter-continental spread of ZIKV remind us of the need for cost-effective, specific, sensitive and technically less-demanding molecular assays to monitor human and vector populations in vulnerable regions. Rapid detection of ZIKV in the field collected mosquitoes assists in optimizing resource allocation for vector control and monitoring the control success. Because ZIKV co-circulates in regions where both dengue and chikungunya viruses are endemic, molecular assays that perform optimally among human and vector specimens play an important role not only in an epidemiological perspective, but also in clinical management, because the infections due to these viruses are often clinically indistinguishable.

## Additional files

Additional file 1: Table S1. Quantification of RNA extracted from field-caught mosquitoes. (DOCX 13 kb)

Additional file 2: Table S2. Cross-reactivity of different primer combinations in the presence/absence of RNA derived from *Aedes* spp., *Culex* spp. and *Anopheles* spp. mosquitoes. (DOCX 15 kb)

Additional file 3: Figure S1. Melting curve analysis demonstrating the non-specific amplification by primer combinations F + R3, F + R4 and F3 + R4 in the presence of mosquito-derived RNA and specificity of F + R3 in a panel of flavivirus and alphavirus RNA. **a**. Melting peak analysis for the F + R4 primer pair **b**. Melting peak analysis for the F3 + R4 primer pair **c.** Melting peak analysis for the F + R3 primer pair. **d**. Melting peak analysis for the F + R3 primer pairs within a panel of flavivirus and alphavirus RNA. *Abbreviation*: NTC, Negative control. (TIFF 2225 kb)

Additional file 4: Figure S2. Melting peak analysis for the rRT-PCR assay performance in field-caught ZIKV-infected mosquitoes. The evaluation is based on the primer combination F2 + R3. The melting peak for ZIKV infected mosquitoes (1–7) and ZIKV non-infected mosquito species (8–14) falls within the same range as the positive control (ATCC® VR-84). (TIFF 679 kb)

## Abbreviations

DENV: Dengue virus
PFU: Plaque forming units
rRT-PCR: real-time reverse transcription polymerase chain reaction
ZIKV: Zika virus
