# Supplementary material for: SYBR green-based one step quantitative real-time polymerase chain reaction assay for the detection of Zika virus in field-caught mosquitoes
Source: Parasit Vectors. 2017 Sep 19;10:427. doi: 10.1186/s13071-017-2373-4 (PMC5604287; doi:10.1186/s13071-017-2373-4)
Supplement: Supplementary file 1 — Quantification of RNA extracted from field-caught mosquitoes. (DOCX 13 kb) [file 13071_2017_2373_MOESM1_ESM.docx]

**Additional file 1: Table S1.** Quantification of RNA extracted from field-caught mosquitoes. Mosquito-derived RNA was extracted by using the QIAamp viral RNA mini kit (Qiagen, Hilden, Germany) according to manufacturer’s instructions. The samples were not spiked with ZIKV. Extracted RNA was quantified by using Qubit RNA Assay kit (Invitrogen, Carlsbad, CA, USA). The quantity of RNA extracted from a DENV-2 culture is given as a comparison.

| **Sample description** | **Mosquito species** | **RNA concentration (ng/ml)** |
| --- | --- | --- |
| Field-caught whole mosquitoes | *Ae. aegypti* | 206 |
|  | *Ae. albopictus* | >1000 |
|  | *Cx. tritaeniorhynchus* | >1000 |
|  | *Cx. sitiens* | >1000 |
|  | *An. Sinensis* | >1000 |
| Head and thorax of field-caught mosquitoes | *Ae. albopictus* | 102 |
|  | *Ae. albopictus* | 82.2 |
|  | *Culex.spp* | 280 |
|  | *Culex.spp* | 117 |
|  | *Ae. aegypti* | 96.2 |
|  | *Ae. aegypti* | 103 |
|  | *Ae. aegypti* | 144 |
| Virus culture | DENV-2  (10^4^ PFU/ml) | >1000 |
