# Supplementary material for: SYBR green-based one step quantitative real-time polymerase chain reaction assay for the detection of Zika virus in field-caught mosquitoes
Source: Parasit Vectors. 2017 Sep 19;10:427. doi: 10.1186/s13071-017-2373-4 (PMC5604287; doi:10.1186/s13071-017-2373-4)
Supplement: Supplementary file 2 — Cross-reactivity of different primer combinations in the presence/absence of RNA derived from Aedes spp., Culex spp. and Anopheles spp. mosquitoes. (DOCX 15 kb) [file 13071_2017_2373_MOESM2_ESM.docx]

**Additional file 2: Table S2.** Cross reactivity of different primer combinations in the presence of mosquito RNA. The primers were tested for the specificity in the presence of only mosquito RNA. The mosquito RNA was not spiked with ZIKV RNA. A sample was classified as cross-reactive when the quantification cycle (C_q_) values were less than 35 and the melting temperature (Tm) peak falls within the range of 78.5 °C to 83 °C

| **Mosquito species** | **Primer pair** | | | |
| --- | --- | --- | --- | --- |
|  | **F + R3** | **F + R4** | **F2 + R3** | **F3 + R4** |
| *Aedes aegypti* | Non-reactive  C_q_=35; Tm=72.4 | **Cross-reactive**  C_q_**=30.7; Tm=82.1** | Non-reactive  C_q_; Tm=74.9 | Non-reactive  C_q_=35; Tm=80.1 |
| *Aedes albopictus* | **Cross-reactive**  C_q_**=32.6; Tm=80.2** | **Cross-reactive**  C_q_**=32.5; Tm=81.4** | Non-reactive  C_q_=35; Tm=74.9 | **Cross-reactive**  C_q_**=31.3; Tm=82.7** |
| *Culex tritaeniorhynchus* | **Cross-reactive**  C_q_**=29.9; Tm=79.5** | Non-reactive  (No amplification signal) | Non-reactive  (No amplification signal) | Non-reactive  C_q_=35; Tm=75.7 |
| *Culex sitiens* | Non-reactive  C_q_=34.2; Tm=71.8 | Non-reactive  C_q_=35; Tm=73.9) | Non-reactive  (No amplification signal) | Non-reactive  (No amplification signal) |
| *Anopheles sinensis* | **Cross-reactive**  C_q_**=32.7; Tm=78.8** | Non-reactive  C_q_=35; Tm=80.45 | Non-reactive  C_q_=35; Tm=78.06 | **Cross-reactive**  C_q_**=32; Tm=79.6** |
| Negative Control | Non-reactive  C_q_=35; Tm=72.1 | Non-reactive  (No amplification signal) | Non-reactive  (No amplification signal) | Non-reactive  (No amplification signal) |
