# Supplementary figures and images for: SYBR green-based one step quantitative real-time polymerase chain reaction assay for the detection of Zika virus in field-caught mosquitoes
Source: Parasit Vectors. 2017 Sep 19;10:427. doi: 10.1186/s13071-017-2373-4 (PMC5604287; doi:10.1186/s13071-017-2373-4)

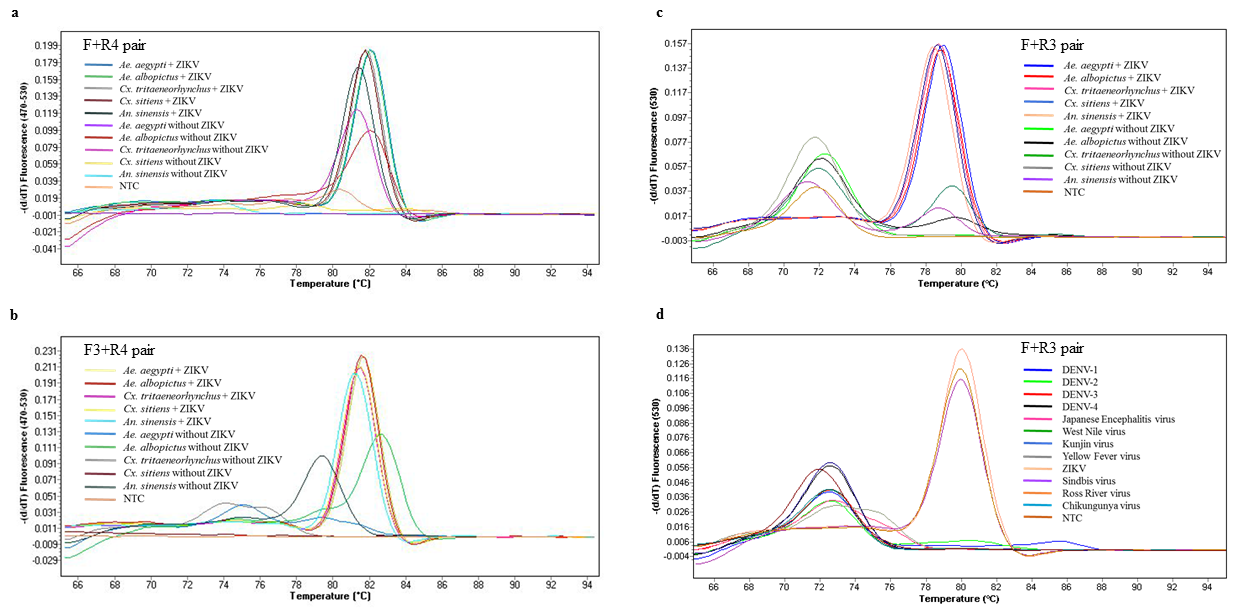

Supplement: Supplementary file 3 — Melting curve analysis demonstrating the non-specific amplification by primer combinations F + R3, F + R4 and F3 + R4 in the presence of mosquito-derived RNA and specificity of F + R3 in a panel of flavivirus and alphavirus RNA. a. Melting peak analysis for the F + R4 primer pair b. Melting peak analysis for the F3 + R4 primer pair c. Melting peak analysis for the F + R3 primer pair. d. Melting peak analysis for the F + R3 primer pairs within a panel of flavivirus and alphavirus RNA. Abbreviation: NTC, Negative control. (TIFF 2225 kb) [file 13071_2017_2373_MOESM3_ESM.tif]

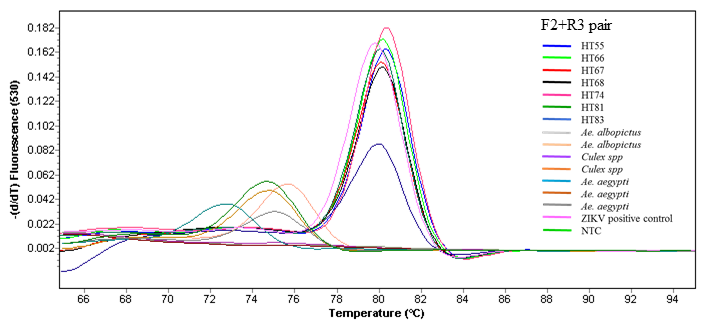

Supplement: Supplementary file 4 — Melting peak analysis for the rRT-PCR assay performance in field-caught ZIKV-infected mosquitoes. The evaluation is based on the primer combination F2 + R3. The melting peak for ZIKV infected mosquitoes (1–7) and ZIKV non-infected mosquito species (8–14) falls within the same range as the positive control (ATCC® VR-84). (TIFF 679 kb) [file 13071_2017_2373_MOESM4_ESM.tif]
